# Socioeconomic inequalities in the prevalence and perceived dangerousness of SARS-CoV-2 infections in two early German hotspots: findings from a seroepidemiological study

**DOI:** 10.1186/s13104-021-05784-5

**Published:** 2021-09-26

**Authors:** Benjamin Wachtler, Stephan Müters, Niels Michalski, Carmen Koschollek, Stefan Albrecht, Sebastian Haller, Osamah Hamouda, Claudia Hövener, Jens Hoebel

**Affiliations:** 1 Division of Social Determinants of Health, Department of Epidemiology and Health Monitoring, Robert Koch Institute, Nordufer 20, 13353 Berlin, Germany; 2 Department of Infectious Disease Epidemiology, Robert Koch Institute, Seestraße 10, 13353 Berlin, Germany

**Keywords:** SARS-CoV-2, COVID-19, Social determinants, Social epidemiology, Seroepidemiological study

## Abstract

**Objective:**

Evidence on socioeconomic inequalities in infections with the novel coronavirus (SARS-CoV-2) is still limited as most of the available studies are ecological in nature and individual-level data is sparse. We therefore analysed individual-level data on socioeconomic differences in the prevalence and perceived dangerousness of SARS-CoV-2 infections in local populations. Data were obtained from a population-based seroepidemiological study of adult individuals in two early German SARS-CoV-2 hotspots (n = 3903). Infection was determined by IgG antibody ELISA, RT-PCR testing and self-reports on prior positive PCR tests. The perceived dangerousness of an infection and socioeconomic position (SEP) were assessed by self-reports. Logistic and linear regression were applied to examine associations of multiple SEP measures with infection status and perceptions of dangerousness.

**Results:**

We found no evidence of socioeconomic inequalities in SARS-CoV-2 infections by education, occupation, income and subjective social status. Participants with lower education and lower subjective social status perceived an infection as more dangerous than their better-off counterparts. In successfully contained local outbreaks of SARS-CoV-2 in Germany, infections may have been equally distributed across the socioeconomic spectrum. But residents in disadvantaged socioeconomic groups might have experienced a higher level of mental distress due to the higher perceived dangerousness of an infection.

## Introduction

Socioeconomic inequalities in ill-health are well described for many non-communicable diseases in various countries [1]. Similar inequalities may emerge in the communicable coronavirus disease 2019 (COVID-19), as the risk for an infection with the novel coronavirus (SARS-CoV-2) may be increased among people in poorer living and working conditions. For instance, living in crowded households may increase the risk for a SARS-CoV-2 infection [2]. Essential workers, e.g. health-care workers or those in the logistic, retailing or public transport sector, might not have the opportunity to effectively protect themselves against virus transmissions. Those workers generally tend to fall into the middle to low income groups [3], whereas the possibility to work from home, a recommended measure to reduce virus transmissions, is more often available to people on higher salaries and with higher qualifications [4]. A modelling study from the USA found that a higher COVID-19 incidence in people from lower-income areas might partly be explained by less opportunities to reduce mobility during the pandemic—presumably due to the lack of opportunity to work from home. Additionally, people from lower-income areas tended to visit more crowded places and were therefore more exposed to potential virus transmissions [5]. Considering that people in lower socioeconomic groups have a higher burden of pre-existing health conditions and may have reduced immune function, they might have an increased susceptibility to SARS-CoV-2 and an increased risk for a severe course of COVID-19 [6, 7].

Empirical evidence of socioeconomic inequalities in the current COVID-19 pandemic is emerging but still sparse [7, 8]. The available evidence is predominantly based on ecological studies from the USA and the UK and suggests that socioeconomically disadvantaged groups have a higher risk of infection with SARS-CoV-2 and severe COVID-19 [8]. But the exclusive use of area-level socioeconomic indicators in most of these studies might underestimate inequalities and findings may be subject to potential ecological fallacy. To date, only few studies can draw on data including socioeconomic information on an individual level [9]. Our analysis therefore used data from a German seroepidemiological study with individual-level data on both infection status and socioeconomic indicators. The study was conducted in the municipalities of Kupferzell in the German federal state of Baden-Wurttemberg and Bad Feilnbach in Bavaria [10]. In Kupferzell, most transmissions of SARS-CoV-2 started with infected inhabitants returning from Northern Italy at the end of February 2020 subsequently visiting a local church concert in early March resulting in a SARS-CoV-2 outbreak. In Bad Feilnbach, early infections were related to local festivities and an outbreak in a nursing home in spring 2020. We analysed if there were socioeconomic inequalities in (1) infections with SARS-CoV-2 and (2) the inhabitants’ perceived dangerousness of an infection.

## Main text

### Methods

The Corona Monitoring Local (CoMoLo) study investigates the seroprevalence of SARS-CoV-2 antibodies in different hotspots in Germany. For this cross-sectional study, a population register-based random sample of the adult population of each municipality was invited to visit a local study centre, where a blood sample and an oropharyngeal swab were taken to examine for antibody prevalence (IgG-ELISA) and acute infection (RT-PCR). Participants answered a short paper-based questionnaire on-site and a detailed questionnaire later on, online or by telephone. Overall response rates were 63% for Kupferzell (20.05.–09.06.2020) and 59% for Bad Feilnbach (23.06.–04.07.2020). Further details can be found elsewhere [10, 11].

We defined an infection with SARS-CoV-2 if either the participant was tested seropositive for SARS-CoV-2 IgG antibodies (Euroimmun SARS-CoV-2-S1 IgG-ELISA: ratio ≥ 1.1), had a positive SARS-CoV-2-RT-PCR swab test during the study or self-reported a positive PCR test prior to the study. The perceived dangerousness of a SARS-CoV-2 infection was assessed by asking the participants: ‘How would you perceive an infection with the novel coronavirus for yourself?’ with a 7-point response scale from 1 (‘completely harmless’) to 7 (‘extremely dangerous’). Individual information on education, occupation, income and subjective social status (SSS) were used as indicators of SEP. Using the 2011 version of International Standard Classification of Education (ISCED), participants’ highest school and vocational qualifications were classified into low (ISCED 1–2), middle (ISCED 3–4) and high (ISCED 6–8) educational levels. Occupational status was assessed with the International Socio-Economic Index of Occupational Status (ISEI) [12]. Equivalised disposable household income was calculated using the OECD-modified equivalence scale. SSS was measured using the MacArthur Scale, a pictorial presentation of a ten rung social ladder on which participants rate their position in society [13].

Prevalence of SARS-CoV-2 infection and mean values of perceived dangerousness ratings were calculated among the 3,903 participants who responded to the detailed questionnaire after their on-site study participation. Logistic and linear regression models were fitted to examine associations of each socioeconomic indicator with infection status and perceived dangerousness. Adjustments were made for age, sex, (education), migrant status, household size and municipality. Weighting factors were used to adjust the net samples of each municipality by age, sex and education to match the official populations statistics. The analysis was performed using Stata version 15.1 (StataCorp LP, College Station, TX, USA) survey data commands.

### Results

Table 1 shows the characteristics of the study population. Population-weighted mean age was 50.0 years (Kupferzell: 49.2; Bad Feilnbach: 50.9). The overall prevalence of a previous or active SARS-CoV-2 infection was 10.4% (95%-CI 9.3–11.6), with 11.8% (95%-CI 10.2–13.6) in Kupferzell and 9.0% (95%-CI 7.6–10.7) in Bad Feilnbach. We found no evidence for differences in the prevalence and adjusted odds ratios (OR) of a SARS-CoV-2 infection between socioeconomic groups, irrespective of the SEP measure used (Table 2): In the low education group, the prevalence was 10.6% (95%-CI 7.5–14.8%) compared to 10.2% (95%-CI 8.6–12.1%) in the high education group (low income 9.6% (95%-CI 7.2–12.7%) vs. high income 9.9% (95%-CI 7.6–12.9%); low occupational status 11.9% (95%-CI 9.0–15.4%) vs. high occupational status 10.5% (95%-CI 8.1–13.6%); low SSS 9.4% (95%-CI 7.0–12.4%) vs. high SSS 9.9% (95%-CI 8.2–11.8%). When only focusing on the results for Bad Feilnbach, we found that for each SEP measure the low SEP groups had slightly higher prevalences than the high SEP groups (Table 2). However, there was no evidence against the null hypothesis of there being no difference.

**Table 1:** Characteristics of the study population (n = 3903)

	Kupferzell n (%)^a^	Bad Feilnbach n (%)^a^
Sex		
Women	1036 (48.5)	1042 (50.8)
Men	932 (51.5)	893 (49.2)
Missing	0 (–)	0 (–)
Age group		
18–39 years	856 (34.8)	629 (30.7)
40–59 years	621 (36.0)	717 (36.5)
60 + years	491 (29.2)	589 (32.8)
Missing	0 (–)	0 (–)
Education		
Low	208 (13.4)	173 (10.9)
Middle	1052 (54.3)	1016 (54.4)
High	706 (32.4)	745 (34.7)
Missing	2 (–)^e^	1 (–)^e^
Occupational status^c^		
Quintile 1 (low)	256 (15.5)	258 (15.5)
Quintiles 2–4	792 (38.2)	674 (32.4)
Quintile 5—(high)	282 (12.2)	287 (13.0)
Missing^b^	638 (34.1)	716 (39.1)
Income^c^		
Quintile 1 (low)	308 (17.0)	304 (17.6)
Quintiles 2–4	1079 (54.2)	985 (49.8)
Quintile 5 (high)	351 (16.6)	369 (17.1)
Missing	230 (12.2)	277 (15.5)
Subjective social status		
Low (1–4)	369 (21.5)	309 (18.7)
Middle (5–6)	911 (49.1)	852 (47.2)
High (7–10)	603 (29.4)	679 (34.1)
Missing	85 (–)^e^	95 (–)^e^

*n* unweighted number of participants

^a^weighted percentage in brackets

^b^non-working population or missing data

^c^quintiles were calculated for non-missing values

^d^based on self-reports

^e^not included in the multivariate analysis

**Table 2:** Prevalences and odds ratios of a SARS-CoV-2 infection by SEP indicators among adults in two early German hotspots

	Total		*p*-value	Kupferzell		*p*-value	Bad Feilnbach		*p*-value
	% (95% CI)	OR (95% CI)		% (95% CI)	OR (95% CI)		% (95% CI)	OR (95% CI)	
Education									
Low	10.6 (7.5 to 14.8)	0.96 (0.58 to 1.58)	0.874	9.5 (6.1 to 14.6)	0.71 (0.39 to 1.32)	0.284	11.9 (6.9 to 19.8)	1.28 (0.62 to 2.63)	0.506
Middle	10.4 (9.0 to 12.1)	0.97 (0.75 to 1.24)	0.788	12.5 (10.3 to 15.1)	1.03 (0.74 to 1.44)	0.842	8.3 (6.5 to 10.5)	0.86 (0.58 to 1.26)	0.434
High	10.2 (8.6 to 12.1)	Ref		11.3 (8.8 to 14.3)	Ref		9.3 (7.3 to 11.7)	Ref	
Occupational status									
Quintile 1 (low)	11.9 (9.0 to 15.4)	1.12 (0.74 to 1.69)	0.604	12.2 (8.5 to 17.3)	1.06 (0.60 to 1.87)	0.841	11.6 (7.7 to 17.1)	1.10 (0.60 to 2.04)	0.753
Quintiles 2–4	10.5 (8.9 to 12.3)	1.01 (0.71 to 1.44)	0.956	12.1 (9.8 to 14.9)	1.05 (0.64 to 1.72)	0.850	8.5 (6.6 to 10.9)	0.89 (0.54 to 1.48)	0.665
Quintile 5 (high)	10.5 (8.1 to 13.6)	Ref		12.1 (8.4 to 17.1)	Ref		9.0 (6.1 to 13.1)	Ref	
Missing^#^	9.7 (8.0 to 11.7)	0.75 (0.49 to 1.16)	0.195	11.1 (8.6 to 14.2)	0.60 (0.33 to 1.08)	0.090	8.4 (6.3 to 11.3)	0.88 (0.47 to 1.63)	0.677
Income									
Quintile 1 (low)	9.6 (7.2 to 12.7)	0.85 (0.53 to 1.34)	0.474	9.5 (6.7 to 13.4)	0.76 (0.41 to 1.38)	0.363	9.7 (6.1 to 15.1)	0.89 (0.45 to 1.79)	0.748
Quintiles 2–4	10.4 (8.9 to 12.1)	1.05 (0.74 to 1.47)	0.799	12.6 (10.4 to 15.2)	1.06 (0.66 to 1.72)	0.803	8.0 (6.1 to 10.3)	0.94 (0.57 to 1.53)	0.792
Quintile 5 (high)	9.9 (7.6 to 12.9)	Ref		11.1 (7.5 to 16.1)	Ref		8.8 (6.2 to 12.4)	Ref	
Missing	11.9 (9.2 to 15.4)	1.19 (0.76 to 1.85)	0.443	12.2 (8.3 to 17.5)	0.99 (0.52 to 1.89)	0.985	11.8 (8.1 to 16.7)	1.22 (0.65 to 2.30)	0.537
Subjective social status									
Low (1–4)	9.4 (7.0 to 12.4)	0.91 (0.63 to 1.33)	0.634	8.1 (5.6 to 11.5)	0.63 (0.39 to 1.03)	0.068	10.9 (7.0 to 16.6)	1.29 (0.76 to 2.20)	0.346
Middle (5–6)	11.2 (9.6 to 13.0)	1.12 (0.86 to 1.45)	0.400	13.0 (10.7 to 15.8)	1.05 (0.74 to 1.49)	0.783	9.3 (7.2 to 11.8)	1.16 (0.78 to 1.75)	0.465
High (7 to 10)	9.9 (8.2 to 11.8)	Ref		12.0 (9.3 to 15.3)	Ref		8.0 (6.0 to 10.4)	Ref	

*%* prevalence in percent, *CI* confidence interval, *Ref.* reference group, *OR* odds ratios from separate logistic regressions on each socioeconomic variable with adjustments for age, sex, (education), migrant status, household size (and municipality); ^#^, non-working population or missing data

The perceived dangerousness of a SARS-CoV-2 infection differed according to education, income and SSS, with those in the low educational, lower income and lower SSS groups perceiving an infection as more dangerous than those in the high SEP groups (Table 3). The associations with education and SSS were found in both hotpots, that with income was only evident for Kupferzell.

**Table 3:** Perceived dangerousness of a SARS-CoV-2 infection by SEP indicators among adults in two early German hotspots

	Total		*p*-value	Kupferzell		*p*-value	Bad Feilnbach		*p*-value
	M (95% CI)	b (95% CI)		M (95% CI)	b (95% CI)		M (95% CI)	b (95% CI)	
Education									
Low	4.38 (4.20 to 4.56)	0.37 (0.16 to 0.57)	0.001	4.35 (4.10 to 4.61)	0.39 (0.08 to 0.70)	0.013	4.42 (4.16 to 4.68)	0.34 (0.06 to 0.62)	0.017
Middle	3.82 (3.74 to 3.89)	0.06 (− 0.05 to 0.16)	0.291	3.76 (3.65 to 3.86)	0.10 (− 0.06 to 0.25)	0.230	3.87 (3.78 to 3.97)	0.01 (− 0.13 to 0.14)	0.930
High	3.75 (3.67 to 3.84)	Ref		3.64 (3.52 to 3.77)	Ref		3.86 (3.75 to 3.97)	Ref	
Occupational status									
Quintile 1 (low)	3.71 (3.57 to 3.84)	0.13 (− 0.06 to 0.32)	0.176	3.72 (3.52 to 3.92)	0.17 (− 0.10 to 0.45)	0.223	3.69 (3.50 to 3.88)	0.08 (− 0.19 to 0.34)	0.569
Quintiles 2–4	3.55 (3.47 to 3.63)	0.04 (− 0.10 to 0.18)	0.594	3.52 (3.41 to 3.63)	0.06 (− 0.15 to 0.27)	0.563	3.59 (3.48 to 3.69)	0.01 (− 0.19 to 0.21)	0.956
Quintile 5 (high)	3.55 (3.43 to 3.68)	Ref		3.43 (3.23 to 3.62)	Ref		3.67 (3.51 to 3.84)	Ref	
Missing^#^	4.35 (4.25 to 4.44)	0.31 (0.13 to 0.49)	0.001	4.28 (4.14 to 4.42)	0.34 (0.06 to 0.63)	0.019	4.40 (4.28 to 4.52)	0.28 (0.05 to 0.51)	0.018
Income									
Quintile 1 (low)	3.93 (3.78 to 4.07)	0.19 (0.01 to 0.37)	0.038	3.91 (3.69 to 4.14)	0.37 (0.09 to 0.65)	0.009	3.94 (3.75 to 4.13)	0.02 (− 0.21 to 0.25)	0.877
Quintiles 2–4	3.88 (3.81 to 3.95)	0.19 (0.06 to 0.32)	0.004	3.87 (3.77 to 3.97)	0.29 (0.10 to 0.48)	0.003	3.90 (3.80 to 4.00)	0.09 (− 0.08 to 0.26)	0.313
Quintile 5 (high)	3.56 (3.44 to 3.68)	Ref		3.36 (3.19 to 3.53)	Ref		3.76 (3.60 to 3.92)	Ref	
Missing	4.06 (3.91 to 4.22)	0.21 (0.03 to 0.39)	0.025	3.91 (3.66 to 4.16)	0.22 (− 0.06 to 0.50)	0.127	4.18 (3.99 to 4.37)	0.18 (− 0.06 to 0.41)	0.138
Subjective social status									
Low (1–4)	4.15 (4.02 to 4.28)	0.42 (0.27 to 0.57)	0.000	4.13 (3.94 to 4.32)	0.48 (0.27 to 0.70)	0.000	4.17 (3.99 to 4.35)	0.35 (0.14 to 0.57)	0.001
Middle (5–6)	3.89 (3.81 to 3.97)	0.18 (0.07 to 0.29)	0.001	3.84 (3.73 to 3.95)	0.24 (0.08 to 0.41)	0.003	3.95 (3.84 to 4.05)	0.12 (− 0.03 to 0.27)	0.105
High (7–10)	3.61 (3.52 to 3.70)	Ref		3.46 (3.33 to 3.59)	Ref		3.74 (3.62 to 3.85)	Ref	

*M* mean, *CI* confidence interval, *Ref.* reference group, *b* coefficients from separate linear regressions on each socioeconomic variable with adjustments for age, sex, (education), migrant status, household size (and municipality); ^#^, non-working population or missing data

### Discussion

This is the first study from Germany that analysed associations of multiple SEP indicators with SARS-CoV-2 infections and perceptions of dangerousness on an individual level. In two population-based random samples from successfully contained early hotspots, we found no evidence for a socioeconomic gradient in SARS-CoV-2 infections. However, local residents with lower education and lower SSS perceived a SARS-CoV-2 infection as more dangerous than their better-off counterparts.

These findings are in line with seroepidemiological studies in the German city of Bonn and the German hotspot region of Tirschenreuth, which reported no educational differences in the risk of SARS-CoV-2 infection [14, 15], and with nationwide seroepidemiological results from Spain that showed no differences in infections according to census-tract income [16]. But they contrast with results from England and the USA that found higher seropositivity among people living in more deprived areas [17] and those with lower education [18]. However, as these results are based on samples of the general population with a much lower prevalence, the comparability to our findings is limited. In contrast to our results, ecological studies that used area-level socioeconomic indicators have found a higher infection risk in districts with less socioeconomic deprivation in Germany [19] for the early stages of the pandemic in March and April 2020 but higher infection risks in more deprived districts during the second wave of the pandemic [20].

Virus spread in early hotspots started in particular social networks, such as visitors of a church concert in Kupferzell, their families and friends. These initial transmissions led to new subsequent infections within the community that affected a broader social spectrum of the local population. However, these transmission networks might as well be more socioeconomically homogenous.

Little is known so far about socioeconomic inequalities in the perceived dangerousness of a SARS-CoV-2 infection. Perceiving an infection as more dangerous might lead to higher stress levels and therefore potentially higher susceptibility to an infection. Furthermore, higher perceived dangerousness might lead to higher levels of depression and anxiety and hence aggravate socioeconomic inequalities in mental health [21]. In contrast, a higher perceived dangerousness might lead to an increased engagement in preventive behaviours [22], and thereby reduce the infection risk. Further research is needed to better understand the possible effects of perceived dangerousness of a SARS-CoV-2 infection on physical and mental health and health disparities.

A strength of this study is that SARS-CoV-2 infections were not only assessed by self-reports, but were also determined by serological and PCR testing, which enabled the detection of both known and yet undetected infections. This is highly relevant, because infections are often mild or asymptomatic and thus may be unknown to infected individuals.

Altogether, the findings suggest that—in early and successfully contained local outbreaks of SARS-CoV-2—infections may be equally distributed across the socioeconomic spectrum, but residents with lower SEP may have experienced a higher burden of mental distress. In order to get a more comprehensive understanding of the social determinants of SARS-CoV-2 infections, data including various measures of individual SEP is needed, ideally for different waves and stages of the epidemic and on a national level.

## Limitations

As our study population was restricted to adult residents in two rural municipalities in Southern Germany with certain events of viral overdispersion in the early stages of the pandemic, the results should be interpreted exclusively within this context and cannot be generalised to the German general population. It should further be considered that these early hotspots were located in socioeconomically less-deprived regions and that their local populations were relatively homogeneous in socioeconomic terms. Findings might differ in other settings and at the national level. Moreover, our findings are limited to the infection risk and the perceived dangerousness of an infection. Even if the infection risk seems to be equally distributed across different socioeconomic groups in our study settings, other outcomes like hospitalisations and mortality linked to COVID-19 could still be associated with a lower SEP, as it is known that risk factors are more frequent in socioeconomically more disadvantaged groups [6]. Further research is needed to better understand these associations and arrive at a more comprehensive picture of health inequalities during the COVID-19 pandemic.

## Data Availability

The data used in this study are not publicly available at this time due to data protection regulations. However, access can be requested for scientific reasons. Requests should be submitted to the Research Data Centre at the Robert Koch Institute, Nordufer 20, 13353 Berlin, Germany. E-mail: fdz@rki.de.

## Abbreviations

COVID-19: Coronavirus disease 2019
ELISA: Enzyme-linked immunosorbent assay
IgG: Immunoglobulin G
ISCED: International standard classification of education
ISEI: International Socio-Economic Index of Occupational Status
OECD: Organisation for Economic Co-operation and Development
PCR: Polymerase chain reaction
RT-PCR: Reverse transcription polymerase chain reaction
SARS-CoV-2: Severe acute respiratory syndrom coronavirus type 2
SEP: Socioeconomic position
SSS: Subjective social status
